# Combined multidisciplinary in/outpatient rehabilitation delays definite nursing home admission in advanced Parkinson’s disease patients

**DOI:** 10.3389/fneur.2023.1128891

**Published:** 2023-04-13

**Authors:** Elien Steendam-Oldekamp, Nico Weerkamp, Judith M. Vonk, Bastiaan R. Bloem, Teus van Laar

**Affiliations:** ^1^Department of Neurology, University Medical Center Groningen, University of Groningen, Groningen, Netherlands; ^2^Excellent Klinieken, Department of Neurology, Bronovo Medical Center, The Hague, Netherlands; ^3^Department of Epidemiology, University Medical Center Groningen, University of Groningen, Groningen, Netherlands; ^4^Department of Neurology, Radboud University Medical Center, Donders Institute for Brain, Cognition and Behaviour, Nijmegen, Netherlands

**Keywords:** Parkinson’s disease, multidisciplinary, rehabilitation, ADL (activities of daily life), nursing home

## Abstract

**Introduction:**

Advanced Parkinson’s disease (aPD) patients have a high risk on definite nursing home admission. We analyzed the effectiveness of an in-and outpatient multidisciplinary rehabilitation, focusing on activities of daily living (ADL) and delaying definite nursing home admission.

**Methods:**

This study included 24 aPD patients, who received a 6-week inpatient multidisciplinary rehabilitation program, including optimization of pharmacotherapy, which was followed by an individualized outpatient support program during 2 years (intervention group). A non-randomized matched control group (*n* = 19), received care as usual. Primary endpoints consisted of the Amsterdam Linear Disability Scale (ALDS) and percentage of patients being able to live independently at home after 2 years. Secondary endpoints included changes in medication (LEDD), motor performance (SCOPA-SPES), cognition (SCOPA-COG), hallucinations (NPI) and depression (BDI).

**Results:**

Overall, 83% of patients were able to return home after the 6-week inpatient intervention, and 65% still lived at home at 2 years follow-up. Median ALDS scores after 2 years in the intervention group were significantly better, compared to the control group (*p* = 0.002). All secondary endpoints had improved significantly vs. baseline directly after the 6-week inpatient rehabilitation, which had disappeared at 2 years follow-up, with the exception of the daily dose of medication, which was significantly higher in the intervention group.

**Conclusion:**

This 2-year follow-up study showed that a combined multidisciplinary in/outpatient rehabilitation program for aPD patients, was able to stabilize ADL functions, and finally delayed definite nursing home admissions in 65% of treated patients.

**Trial registration:**

filenumber M10.091051; ABR code NL32699.042.10.

## Introduction

Parkinson’s disease (PD) has an enormous impact on quality of life (1–3). Many patients lose the ability to live independently at home (3–6). Important reasons for nursing home admission include cognitive deterioration, hallucinations, older age and the loss of activities of daily life (7–12). PD patients in nursing homes are the most expensive group to treat, increasing the overall costs by approximately 4 times the costs of living at home (13). No intervention in aPD patients thus far has shown any delay or prevention of nursing home admission.

This study investigated whether a combined multidisciplinary in/outpatient rehabilitation program, focusing on ADL functioning, was able to delay definite nursing home admission. For this purpose, we performed a non-randomized controlled trial during 2 years, to analyze the short-and long-term effectiveness of a 6-week inpatient rehabilitation program, followed by a multidisciplinary support program during 2 years in aPD patients, who were on the brink of losing their independence.

## Materials and methods

### Participants

Recruitment of patients for this prospective, controlled, multicenter trial took place at the outpatient departments of 2 hospitals (Martini hospital/MZH and the University Medical Centre Groningen/UMCG) in the northern part and at seven different nursing homes in the southern part of the Netherlands. Inclusion criteria were: (a) diagnosis of PD according to the UK Brain Bank Criteria (14); and (b) combination of motor-, cognitive-and behavioral problems, interfering with independent living at home, necessitating direct nursing home admission. Exclusion criteria included: (a) presence of atypical Parkinsonism, (b) inability or unwillingness to give informed consent; and (c) unstable general medical conditions, requiring intensive or invasive treatment. We included two groups of aPD patients (Table 1). All of them were not able to live independently at home at the moment of inclusion. The first group consisted of 24 aPD patients, who received an in/outpatient rehabilitation program. The second group consisted of 19 aPD patients, serving as matched controls, who had been admitted to a nursing home, receiving care as usual. Data of controls were selected from a group of nursing home patients in the Southern part of the Netherlands, who had been admitted to a nursing home since 0–3 months. The controls were selected from a larger population of nursing home patients with aPD, that participated in an already completed quality of care study in Dutch nursing homes (15, 16). To avoid selection bias, patients in the intervention group were included sequentially, based on the following order of referral. The ethics committees of the University Medical Center Groningen and University Medical Center St. Radboud gave informed consent for the study.

**Table 1:** Demographics at baseline.

	Intervention PfP group (*n* = 24)	Control group (*n* = 19)
**Patients characteristics**		
Men (*n*/%)	13 (54%)	7 (37%)
Age (years)	70.0 (65.25–77.0)	76.0 (70.0–83.0)
Disease duration (years)	8.0 (4.5–11.0)	9.0 (5.0–12.0)
Hoehn and Yahr stage		
IV (*n*/%)	15 (62.5%)	13 (68.4%)
V (*n*/%)	9 (37.5%)	6 (31.6%)
Daily LEDD** (mg)	1097.5 (568.75–1743.75)	687.5 (400–1,000)
Probable PD dementia (*n*/%)^#^	13 (54.2%)	14 (73.6%)
Visual hallucinations (*n*/%)	10 (41.7%)	3 (15.8%)*
Depression (*n*/%)^##^	6 (25%)	6 (31.6%)
**Primary outcome**		
ALDS score	59.3 (41.2)	69.3(16.0)*
**Secondary outcomes**		
SCOPA-SPES score	23.5 (16.5–29.25)	27.0 (22.0–36.0)
SCOPA-COG score	22.0(14.0–26.75)	15.0 (11.0–20.0)*
NPI score	2.0 (1.0–6.0)	2.0 (1.0–3.0)
BDI score	11.0 (9.75–15.0)	12.0 (11.0–14.0)

Data reflect median scores, with percentages or the Interquartile Range between brackets. Both groups did not show significant differences at baseline (Mann–Whitney test).*Except for ALDS scores (*p* = 0.022) at baseline, which were higher in the controls (corresponding with better performance), cognition (*p* = 0.013) and presence of hallucinations (*p* = 0.031). **LEDD, levodopa-equivalent daily doses. ^#^SCOPA-COG < 22.^##^BDI > 14.

### Treatment programs of both groups

**Figure 1:**
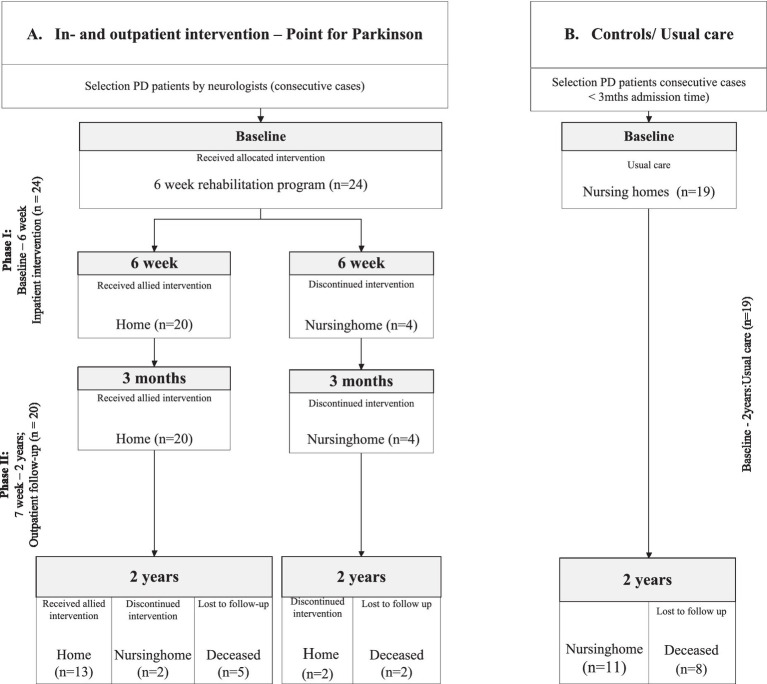
Flowchart of the study divided into: Phase I. The inpatient intervention program at the Groningen expertise center (Point for Parkinson, PfP); Phase II. The outpatient follow-up program (Parkinson specialist/neurologist PfP and ParkinsonNet allied health professionals). **(A)** Intervention Group; who received an inpatient intervention program at the expertise center Point for Parkinson Groningen. **(B)** Control Group; who were directly admitted to a nursing home and received care as usual.

The rehabilitation program consisted of 2 parts, Phase I and II (Figure 1; flowchart). Phase I; the inpatient program during 6 weeks was delivered at the rehabilitation unit of the Parkinson expertise center in Groningen (Point for Parkinson, PfP). The inpatient program consisted of 1 week of baseline observations, including the assessment of motor-, cognitive-and behavioral scales. Thereafter, a medical-and allied health treatment plan was performed during 5 weeks. During this inpatient period 2–3 h physiotherapy, 1 h speech therapy, 1–2 h occupational therapy, 2–4 h of professional coaching, 2 h of social work and 0.5 h dietary support a week were provided, with minimal interindividual variations (Supplementary Table S1).

Their individualized treatment plan, was initiated by the allied health professionals of PfP in phase I, focusing on the presented signs and specific symptoms of each patient. These treatments were provided, according to the national guidelines on allied therapies, and handed over to allied health professionals, trained by the Dutch ParkinsonNet (17, 18), in their own environment during the rest of the follow-up period during phase II (17). The medication adjustments in the intervention group strictly followed the Dutch multidisciplinary guideline on PD, focusing on optimal dosing of motor symptoms, the implementation of advanced therapies if necessary, and adequate treatment of non-motor symptoms (19, 20). Medication adjustments (Supplementary Table S2) took place based on predefined objectives, like improvement of walking or reduction of visual hallucinations. Patients were weekly evaluated by the complete multidisciplinary team, including a neurologist and a specialist elderly care with special training in treating aPD patients, Parkinson nurses, physiotherapists, social workers, speech therapist, occupational therapist and dietician. All dopaminergic medication was converted into levodopa-equivalent-daily doses (LEDD) (19, 21).

The control group received care as usual. Care as usual included adjustments of medication regimens, if considered appropriate by the specialist elderly care (according to the Dutch multidisciplinary guidelines elderly care-Verenso), as well as allied health interventions, mostly on a regular basis, with a focus on retaining functions. Standard care in nursing homes in the Netherlands includes in most cases physiotherapy in 88% of patients, during 0.5–1 h per week and 26.3% follow speech therapy (22, 23). If a patient is admitted to a nursing home, treatment by the neurologist often ends. Neurologists are still involved in the nursing home patients in only 42% of cases.

### Outcome measurements

#### Primary endpoints

We defined two primary endpoints. The first one was the difference between both groups in the change of the Amsterdam Linear Disability Scale (ALDS) scores between baseline and 24 months. In total 26 items of the ALDS, were selected, hierarchically ordered from simple to more complex. The ALDS is calculated as a logistic regression coefficient, expressed in thetas, which can be transformed linearly into values between 10 and 90, with higher scores indicating better functional ability. ALDS has shown adequate clinometric properties in patients with PD (24, 25) and can be used as a reliable indicator of the functional status of aPD patients in nursing homes as well as in an outpatient setting (26). The other primary endpoint, directly related to the ALDS, was the percentage of patients, discharged from the nursing home setting, living independently in both groups after 2 years.

#### Secondary endpoints

The first secondary endpoint was the change in LEDD during the 2 year follow-up in both groups. Motor performance was assessed using the Short Parkinson’s Evaluation Scale (SCOPA-SPES). The SCOPA-SPES is a reliable and valid instrument to assess motor functions (27, 28). The SCOPA-SPES is a 21-item scale with 3 sub-groups, being Motor Evaluation, ADL, and Motor complications. Higher scores on the SCOPA-SPES reflect more severe motor impairments.

The cognitive status of all patients was assessed with the SCOPA-COG, a short, reliable, and valid instrument that examines 4 cognitive domains: memory, attention, executive functioning and visuospatial functioning (29, 30).

The Neuropsychiatric Inventory Questionnaire (NPI-Q) (30, 31). The NPI-Q contains 12 neuropsychiatric symptoms, measuring the presence of items (yes/no), the severity by a 3-point scale and the impact of the symptoms by a 6-point scale, with a maximum overall score of 36. All secondary endpoints analyzed the difference in change over 24 months between both groups.

Mood changes were measured using the Beck Depression Scale (BDI), which is the most valid instrument to measure depression in Parkinson’s disease (31–34). This 21-item questionnaire has a 4-point scale, whereby a total score of >14 indicates depression in a PD population (32).

Behavioral and neuropsychiatric disturbances were evaluated using the Neuropsychiatric (35, 36).

In order to get an impression of the acute and subacute effects of the inpatient intervention, all primary and secondary endpoints were also evaluated at 6 and 12 weeks.

#### Statistical analysis

Previous Dutch data suggested an average yearly decline of 1.3 point in ALDS in patients with PD (37) To detect this difference with 80% power and an α of 5%, assuming a standard deviation of 8.9 (24) and a correlation of 0.75 between the measurements, a sample size of at least 19 patients for both groups was calculated. The type 1 probability failure testing this null hypothesis is 0.05. Assuming a drop-out of up to 25% in the intervention group, we aimed to include 24 patients. Based on these data, we postulated that a change in ALDS score of ≥3 points would represent a clinically relevant difference at 2 years.

SPSS 23 was used to perform the statistical analyses. Regression analysis and analysis of covariance were performed to correct for the difference of the ALDS and SCOPA-COG scores at baseline, whereby control patients had a significant better ALDS score at baseline compared to the intervention patients, whereas intervention patients performed better on SCOPA-COG at baseline. The Mann–Whitney test was used to calculate the between-group differences of baseline vs. 2-year follow-up scores. The Wilcoxon test and Friedman test were used to calculate the changes in endpoint scores over time. Given the small group sizes, 95% confidence intervals (CI) were established also using bootstrapping (*n* = 1,000 bootstraps). To avoid selection bias, patients in the intervention group were included sequentially (consecutive cases), based on the following order of referral. The known variables, like increasing age, functional impairment, Parkinson’s disease dementia (PDD) and hallucinations, were interrogated as potential confounders. Besides these variables also PD medication and disease duration were tagged as potential confounders.

The protocol was approved by the Medical Ethical Committee (METc) of the University Medical Center Groningen (UMCG), using the checklist with TREND criteria (filenumber M10.091051; ABR code NL32699.042.10). The principal investigator TvL was responsible for the integrity of the design, the conduct and analysis of the study.

#### Role of the funding source

The sponsors of this study had no involvement in the study-design, data collection, data analysis and interpretation, neither in writing the final report. Final responsibility for submitting the publication was taken by BRB and TvL.

#### Results

Overall, 43 PD patients were included; 24 patients in the intervention group and 19 patients in the control group with care as usual (Figure 1). The mean duration of admission of patients in the inpatient program was 42 days (SD 10.79).

### Primary outcomes

Amsterdam Linear Disability Scale scores of the patients in the intervention group significantly improved directly after the inpatient intervention (6 weeks), but slightly worsened thereafter over 2 years, resulting in final scores which were comparable to baseline (Supplementary Table S3). Sub-analysis of the intervention group showed a significant difference (37.8 points; *p* = 0.030, 95%CI) in baseline ALDS scores between patients who could return home (higher scores) and those who were admitted to a nursing home (Supplementary Figure S1). Both groups improved on their ALDS scores, however only the group who went home showed a significant improvement (7.5 points. *p* = 0.000, 95%CI).

**Figure 2:**
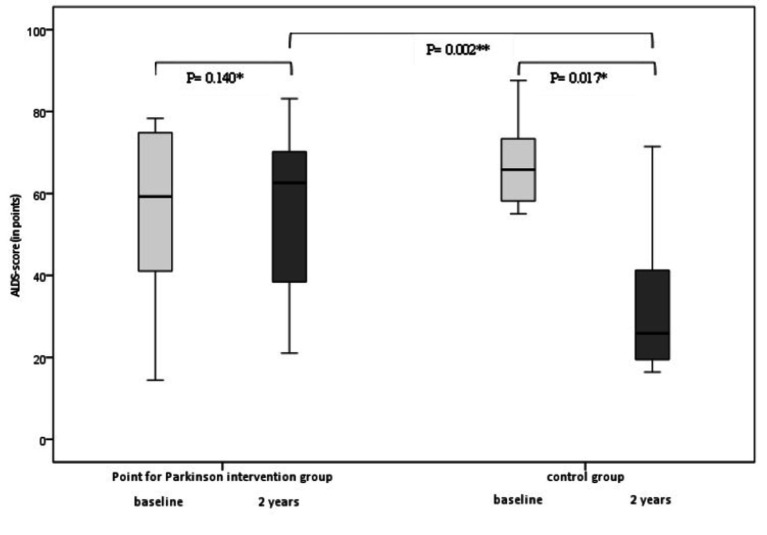
Box plots of median changes in primary outcome (ALDS scores/min-max range) between the intervention group and the control group at baseline (between group differences at 2 years *p* = 0.002 95% CI). *Wilcoxon, **Mann–Whitney *U*.

Amsterdam Linear Disability Scale scores of the intervention group returned again to baseline scores after 2 years (Table 2). On the contrary, the ALDS scores worsened significantly over 2 years in the control group; with 40 points (*p* = 0.017, 95%CI) after 2 years (Figure 2). Regression analysis and analysis of covariance were performed to correct for the difference in ALDS scores between both groups at baseline (controls had better ALDS scores), but both analyses showed a significant worsening of scores in the control group (*p* = 0.002) compared to intervention group.

**Table 2:** Primary and secondary endpoints of patients in the rehabilitation-and control group.

Test score	Intervention group (*n* = 24)			Control group (*n* = 19)			Between group differences at 2 years
	Baseline median (IQR)	2 years median (IQR)	Value of p*	Baseline median (IQR)	2 years median (IQR)	Value of p*	Value of p**
**Primary outcomes**							
ALDS	59.28 (31.09–72.29)	62.61 (34.87–71.99)	0.140	69.33 59.9–75.17	29.33 (18.29–54.72)^##^	0.017	**0.002**
Patients living independently at home (*n*, %)	–	13 (65%)		–	0(0%)		
**Secondary outcomes**							
LEDD (mg)	1097.5 (568,75-1743,75)	1592.5 (1000.0–2105.0)	0.002	678.5 (400–932.5)	820 (400–1,000)	0.144	**0.042**
SCOPA-SPES	23.50 (16.5–29.25)	24.00 (20.0–35.0)	0.462	27.00 (22.0–36.0)	33.00 (29.0–37.0)	0.306	0.141
**Subitems**							
Motor evaluation	9.00 (6.25–13.5)	12.00 (7.0–15.0)	0.786	11.00 (9.0–17.0)	16.00 (13.0–19.0)^##^	0.15	**0.034**
ADL	10.50 (9.0–14.0)	11.0 (8.0–17.0)	0.495	12.00 (9.0–17.0)	16.00 (12.0–17.0)	0.203	0.157
Motor complications	2.00 (0.0–3.75)	4.00 (3.0–5.0)	0.68	2.00 (1.0–3.0)	2.00 (0.0–2.0)	0.216	**0.041**
SCOPA-COG	22.00 (14.0–26.75)	20.00 (11.0–27.0)	0.753	15.00 (11.0–20.0)	9.00 (1.0–17.75)^##^	0.042	0.062
**Subitems**							
Memory	6.00 (4.0–9.0)	5.00 (5.0–7.0)	0.073	5.00 (4.0–7.0)	3.00 (0.0–6.50)	0.216	0.125
Attention	3.00 (1.5–4.0)	2.00 (1.0–4.0)	0.336	3.00 (2.0–4.0)	2.00 (0.0–3.25)	0.066	0.509
Executive functioning	9.00 (4.25–10.0)	9.00 (4.0–11.0)	0.248	5.00 (3.0–6.0)	2.50 (1.0–4.75)^##^	0.167	**0.021**
Visuospatial functioning	4.00 (3.0–5.0)	3.00 (1.0–7.0)	0.339	3.00 (1.0–4.0)	0.50 (0.0–4.25)	0.084	0.168
NPI	2.00 (1.0–6.0)	0.50 (0.0–1.25)^#^	0.048	2.00 (1.0–3.0)	0.0 (0.0–8.00)	0.31	0.488
Hallucinations (*n*/%)	10(42%)	5 (38%)	0.753	3 (16%)	5 (45%)	0.18	0.391
Depression (*n*/%)	7 (29%)	1 (8%)	0.157	9 (47%)	4 (36%)	0.564	0.339
Delusions(*n*/%)	2 (8.3%)	0	0.317	0	2 (18%)	0.317	0.083
BDI	11(9.75–15.0)	12(11.0–14.0)	0.18	12(11.0–14.0)	11.5 (7.5–14.5)	0.345	0.685

Scores are median scores and range IQR25-IQR75, 95% CI. ^#^Suggest improvement over time. ^##^Suggest worsening over time.^*^Wilcoxon.^**^Mann–Whitney.Bold values: significant between group differences at 2 years follow-up.

Overall 20 out of 24 patients from the intervention group could return home after the 6-week inpatient program (83.3%). All 20 patients still lived independently at 3 months follow-up, and 13 patients (65%) even lived still independently at home after 2 years follow-up. All control patients remained in the nursing home throughout the follow-up period. After 2 years, seven patients in the intervention group and eight patients in the control group had died.

### Secondary endpoints

The median LEDD of the intervention group increased significantly with 495 mg (*p* = 0.002, 95%CI) at the end of the intervention. LEDD in the control group increased non-significantly with 141.5 mg (*p* = 0.144, 95%CI). Subcutaneous infusion of apomorphine was initiated in 3 patients in the intervention group, and the dose of already existing apomorphine infusion was increased in 2 patients (mean 506.25 mg LEDD). Overall dopaminergic medication increased in 75% (*n* = 18) of patients by a median LEDD of 495.00 mg, whereas in only 4 patients (17%) dopaminergic medication was decreased and in 2 patients (8%) their original baseline dose was continued in the intervention group. The level of cholinesterase inhibitors, atypical antipsychotics and tricyclic antidepressants also showed a significant difference between both groups (Supplementary Table S2).

During phase II, physiotherapy was applied significantly more frequent, compared to baseline, with an increase of 29.2% (*p* = 0.025). The other allied health therapies in phase II were not significantly increased vs. baseline; [speech therapy 13.3%, (*p* = 0.317), occupational therapy 15.8% (*p* = 0.083), social work 5% (*p* = 0.317) and dietary support 5% (*p* = 0.317)].

The SCOPA-SPES scores in the intervention group worsened 0.5 points, whereas the control group worsened 5 points over 2 years, which however was not significantly different (Table 2).

Sub-analysis of the intervention data showed a significant improvement of the SCOPA-SPES after 6 weeks, which worsened again during 2 years follow-up (Supplementary Table S3). The motor complication scores, being part of the total SCOPA-SPES, showed the longest improvement, lasting up to at least 3 months.

The SCOPA-COG scores in the intervention group did not change significantly over 2 years (Table 2). However, the control group showed a SCOPA-COG decrease of 6 points (*p* = 0.042, 95%CI). The most important cognitive change between both groups was related to worsening of the executive functioning (*p* = 0.021) in the control group.

Regression analysis and analysis of covariance were performed to correct for the difference in SCOPA-COG scores between both groups at baseline, but both groups showed the same significant worsening. After joining the intervention program sub-analysis showed that the median score of the SCOPA-COG improved significantly with 6 points (*p* = 0.000, 95%CI), which remained stable in phase II at 3 months (27.5; IQR 20.25–30.75).

NPI scores improved significantly (*p* = 0.001) after phase I in the intervention group (Supplementary Table S2), and this improvement was still significantly different vs. baseline at 3 months (*p* = 0.046). Especially the occurrence of hallucinations decreased significantly from 42 to 25% of the patients in the intervention group (*p* = 0.014). This difference had disappeared at 2 years follow-up.

BDI scores had improved significantly in the intervention group after phase I (Supplementary Table S2), which difference also had disappeared after 2 years follow-up (Table 2).

## Discussion

This study describes the positive outcomes of a long-term, controlled trial on the efficacy of an in/outpatient, multidisciplinary Parkinson rehabilitation program, including medication optimization. Importantly, only aPD patients were included, who were not longer able to live independently at home at the time of inclusion. Our data indicate that a multidisciplinary intervention, is able to keep these aPD patients stable for at least 2 years, as shown by the functional ALDS scores, which was not the case in the controls with care as usual. Moreover, the controls experienced a significant decrease in their functional capability, which is in line with previous data showing that PD patients in a nursing home with moderate cognitive impairment showed deterioration of MDS-ADL scores of 1.78 points in 6 months (38). Post-hoc analysis of the baseline ALDS scores of treated patients indicated that returning home was correlated to higher baseline ALDS scores (Supplementary Figure S1).

Our study reports for the first time that multidisciplinary rehabilitation of aPD patients is able to postpone definite nursing home admission by at least 2 years in 65% of cases. None of the control patients left the nursing home during the same period.

An important difference between both groups was the level of dopaminergic stimulation. The intervention group showed a significant increase in LEDD, including initiation or optimization of continuous infusion therapies in some patients. This suggests that optimal pharmacotherapy offers important advantages on the short-and long-term. The pharmacotherapy for PD patients in nursing homes is suboptimal, with 44% of PD patients being most of the time ‘off’, and low LEDDs, varying from 400 to 500 mg/day, while patients had already PD for at least 7 years (10, 39). In our study the mean LEDD at baseline of the intervention group was 1097.5 mg, but still 75% of included patients were considered to be underdosed.

The other secondary endpoints showed that the effect of the inpatient rehabilitation (phase I) was most optimal directly thereafter, which decreased over time during phase II, resulting in comparable outcomes at 2 years follow-up, except for the NPI, which improved significantly in the intervention group, despite a higher LEDD in the intervention group, which could have increased hallucinations, but that did not happen (40–42). The increased dosages of apomorphine, CHEI’s and clozapine in our study are the most likely explanation for this finding (43–47). This is an important result, because visual hallucinations are the strongest predictor of definite placement in nursing homes (9, 11, 12). The intervention program also included a small group without the need to change their LEDD. These patients also improved on their motor-and ADL scores, stressing the importance of allied therapies and other disciplines involved in our rehabilitation program. These findings strengthen the evidence of the effect of allied therapies within a multidisciplinary rehabilitation treatment (without medication adjustments) on patients with early stage PD on ADL functioning and QoL (48, 49).

Improvement of the cognition is very likely due to optimization of medication. Suppletion of the cholinergic deficit with rivastigmine, eventually in combination with optimization of the levodopa dose. In 54% of the PD patients rivastigmine was added to their medication regimen during the treatment. Overall 87% of the PD patients showed improved scores (SCOPA-COG) with an average of 6 points. This is in line with other studies, which found improvement of cognition after optimized treatment as well (45–47).

This study also has some limitations. Both arms included relatively small numbers of patients, although this was based on our power calculations before study onset. The fact that this small sample already resulted in significantly positive outcomes supports the strength of our rehabilitation concept. We performed bootstrapping to provide a more representative outcome, to control for the relatively small sample size. However, the outcomes after bootstrapping were not significantly different from the original data.

At 2 years follow-up, seven patients in the intervention group (29.2%) and eight patients in the control group (42.1%) had died. This means that the loss-to-follow-up is a potential threat to validity and might cause significant bias (50). A chi-square test was performed to control for these differences and no dissimilarities were found (0.7816; *p* = 0.3766) between the intervention and control group. The differences in ALDS-and SCOPA-COG scores at baseline might also have impacted the effect size. However, regression analysis with adjustment for these baseline values and analysis of covariance with adjustment for the baseline values gave exactly the same output, suggesting there was no serious influence on the effect size.

Another limitation is the lack of QoL data in our control group. This would have offered an extra possibility to discuss the observed functional improvements in this study. Living longer at home and greater independence in activities of daily living provide better QoL in late-stage Parkinson, as was shown previously (3).

Finally, neither patients nor the assessors were blinded with respect to the intervention. This might have influenced the final outcomes. However, the most important finding, being the percentage of patients returning home after the intervention, and still staying at home after 2 years in 65% of cases, cannot be explained by this open assessments, which is also the case for the LEDD changes, which are the result of a particular vision on optimal treatment of aPD, instead of assessment bias. Therefore, the stabilization in the rehabilitation group and the clear worsening in the control group can be considered as real and important effects of our rehabilitation program.

Our findings potentially have large implications. If in/outpatient aPD rehabilitation, with a focus on optimization of pharmacotherapy, is able to stabilize advanced PD patients and delay nursing home admission, many more advanced PD patients should be offered similar programs. This would not only improve their independence but would also significantly save costs of nursing home admission (around 90.000 Euro per patient per year in the Netherlands). A Norwegian study showed that costs arising from nursing home placement were 5 times higher for PD patients compared to controls (51) For exact cost-effectiveness of this particular concept further research is necessary. The costs of the control group are related especially to the costs of institutional care. The costs of the intervention group exist of the inpatient intervention/rehabilitation period and the outpatient support program. The inpatient program costs are estimated on 300 Euro per day, which makes around 12.500 Euro for 6 weeks. The costs of the outpatient program are based on a questionnaire covering the health-care costs during the previous 3 months, including medical care, allied health care, home care nursing as well domestic informal care, and even hospital admissions, if needed. These costs summed up to 4.000 Euro/3 months, which means a yearly cost of 16.000 Euro/patient in the intervention group. If it is hypothesized that those costs were kept at the same level during the 2 year follow-up, this would mean an overall cost of the intervention group of 44.500 Euro vs. 180.000 Euro of the nursing home group. This means that not only the QoL is improved by our intervention, but that the prevention of definite nursing home admission also implicates a huge financial benefit of almost 70.000 Euro per patient per year.

The most important message of our study is the huge benefit of intensive rehabilitation for advanced PD patients. We have shown the impact on living independently at their own houses, which is a significant contribution to their quality of life. Therefore, in every country neurologists should take responsibility to create places where advanced PD patients are rehabilitated, in order to prevent definite nursing home admission. The place where this rehabilitation should take place will differ between countries, and may vary between hospital settings (which is mostly too expensive), rehabilitation centers or, as in our case, specialized nursing homes, creating a setting without time–pressure, but offering a stimulating atmosphere, created by an educated multidisciplinary PD team. Without these rehabilitation options, advanced PD patients will have a reduced quality of life at high costs, because our study also shows the huge financial benefit of delaying definite nursing home admission.

However, before this approach can be recommended to all advanced PD patients or even be more widely used in chronic patients on the brink of nursing home admission, our data have to be confirmed in larger samples, preferably in other clinics, and in other countries, in order to validate our PD rehabilitation concept.

## Data availability statement

The raw data supporting the conclusions of this article will be made available by the authors, without undue reservation.

## Ethics statement

The studies involving human participants were reviewed and approved by Trial registration: filenumber M10.091051; ABR code NL32699.042.10. The patients/participants provided their written informed consent to participate in this study.

## Author contributions

ES-O and TL contributed to the research design. ES-O wrote the first draft of the manuscript. TL and BB were responsible for revisions. ES-O and NW were responsible for data collection. JV was responsible for statistics. All authors made a substantial, intellectual contribution to the work read and approved the final manuscript.

## Funding

This study was supported by the UMCG innovation fund and prof. Van der Valk Stichting.

## Conflict of interest

The authors declare that the research was conducted in the absence of any commercial or financial relationships that could be construed as a potential conflict of interest.

## Publisher’s note

All claims expressed in this article are solely those of the authors and do not necessarily represent those of their affiliated organizations, or those of the publisher, the editors and the reviewers. Any product that may be evaluated in this article, or claim that may be made by its manufacturer, is not guaranteed or endorsed by the publisher.

## Supplementary material

The Supplementary material for this article can be found online at: https://www.frontiersin.org/articles/10.3389/fneur.2023.1128891/full#supplementary-material
