# Supplementary material for: Combined multidisciplinary in/outpatient rehabilitation delays definite nursing home admission in advanced Parkinson’s disease patients
Source: Front Neurol. 2023 Apr 13;14:1128891. doi: 10.3389/fneur.2023.1128891 (PMC10133548; doi:10.3389/fneur.2023.1128891)
Supplement: Supplementary file 1 [file Data_Sheet_1.PDF]

Supplementary Material

Combined multidisciplinary in/outpatient rehabilitation delays definite nursing home admission in advanced Parkinson's disease patients

Elien Steendam-Oldekamp,[1] Nico Weerkamp,[2] Judith M. Vonk[3], Bastiaan R. Bloem,[4] Teus van Laar[1]

Correspondence: Elien Steendam-Oldekamp, University Medical Centre Groningen, Hanzeplein 1, 9700 RB Groningen, The Netherlands.

Email: t.steendam@umcg.nl

Supplements

Therapy	Frequency (/week)	Hours/ week	Intervention type	Goals/targets
Physical therapy(1,2)	4	2-3	Individual and group therapy	1. Improving transfers, posture, reaching and grasping, balance and walking.<br>2. Maintaining and improving physical condition<br>3. Preventing falls<br>4. Improving mobility
Speech therapy(3)	1-2x	1	Individual	1. Dysarthria and communication<br>2. Dysphagia<br>3. Salivation
Occupational therapy(4,5)	1	1.5	Individual	1. Stimulation of self-management and empowerment<br>2. Offering daily structure<br>3. Coping with stress and time pressure<br>4. Improving motor skills<br>5. Improving focused attention<br>6. Applying mindfulness-based cognitive strategies<br>7. Coping with dual tasks<br>8. Applying cues<br>9. Optimisation of the physical habitat<br>10. Counselling of caregivers
Professional activity coach	2-4	2-4	Group therapy	Activities which contribute to physical-, mental- and social well-being of the patients. These activities consist of:<br>1. leisure activities (e.g. music, painting)<br>2. educational activities (e.g. computer lessons, traffic classes)<br>3. ADL stimulating activities (e.g. cooking, shopping)<br>4. practical activities (e.g. gardening, administration)

Social work	On demand (mean 2 hrs./week)	2	Individual and/or t partner/ caregiver	Psychosocial interventions<br>1. Inventory on burden of disease (patient and partner/caregiver)<br>2. Providing information on assistance- and support-networks<br>3. Exploring fields of interest of patient and/or partner/caregiver<br>4. Advise on coping with Parkinson’s disease
Dietician(6)	1-2	0.5	individual	1. weight loss and/or malnutrition<br>2. obstipation<br>3. intake of medication and response fluctuations<br>4. unwanted weight gain or obesity<br>5. chewing and swallowing disorders<br>6. delayed gastric emptying<br>7. orthostatic hypotension<br>8. role of vitamins and minerals

Table 1. Allied professional interventions at PFP 6 week customized intervention program, according the Dutch guidelines.

	Intervention group (n=24)			***Control group (n=19)***			***Between group difference at 2 years***
	**Before intervention median (IQR)**	**After intervention median (IQR)**	**p-value ***	**Before admission median (IQR)**	**After admission median (IQR)**	**p-value ***	**p-value ****
Medication							
**Cholinesterase inhibitors (n/%)** *(Rivastigmine/ Galantamine/ Donepezil)*	0 (0%)	13 (54.2%)	0.001	8 (42.1%)	4 (21.1%)	0.059	**0.011**
**Atypical Antipsychotics (n/%)** *(Clozapine, quetiapine)*	1 (4.2%)	4 (16.7%)	0.035	8 (42.1%)	4 (21.1%)	0.008	0.148
**tricyclic antidepressants (n/%)** *(amitriptyline, nortriptyline)*	0(0%)	4(16.7%)	0.059	4(21.1%)	0(0%)	0. 046	0.065
Allied Therapies (used)							
Physiotherapy (n/%)<br>(mean (SD) minutes a week)	5(20.8%)<br>23.1 (4.0)	10(50%)<br>42.8(10.6)	0.025<br>0.017		18 (94.7%)<br>26.6(1.71)		
Speech therapist (n/%)<br>(mean (SD) minutes a week)	4(16.7%)<br>6.0(1.9)	6(30%)<br>10(3.8)	0.317<br>0.136		5(26.3%)<br>9.5(4.0)		
Occupational Therapy (n/%)	1(4.2%)	4(20%)	0.083		-		

(mean (SD) minutes a week)	7.05 (7.1)	12.5(7.7)	0.102	
Dietician				
(n/%)	0(0%)	1(5%)	0.317	-
(mean (SD) minutes a week)	0	1.88 (1.9)	0.317	
Social work				
(n/%)	0(0%)	1(5%)	0.317	-
(mean (SD) hours a week)	0	0.25(0.25)	0.317	

Table 2: Medication median scores and IQR's=IQR75-IQR25, 95% CI). Allied therapies n= amount of patients and percentage. Mean minute/hours per patients per week with SD

* Wilcoxon

** Mann-Whitney

Cholinesterase inhibitors (CHEI) were started in 13 patients (54.2%) in the intervention group, whereas in the control group cholinesterase inhibitors were stopped in 4 patients (21.1%). Atypical antipsychotics were started in 3 patients (12.5%) of the intervention group, whereas atypical antipsychotics were stopped in 4 patients (21.1%) in the control group. Four patients (16.7%) of the intervention group received tricyclic antidepressants (TCAs) during the inpatient phase of the intervention, whereas in 4 patients (21.1%) of the control group TCAs were stopped.

**Scores Intervention PfP group**							
Test score	**Baseline median(IQR)**	**6 weeks median(IQR)**	**p-value ***	**3 months median(IQR)**	**p-value ***	**2 years median(IQR)**	**p-value ****
Primary outcome							
**ALDS**	59.28 (31.09-72.29)	69.89# (58.05-78.34)	**0.000**	70.03# (50.21-80.46)	**0.005**	62.61 (34.87-71.99)	0.140
Secondary outcomes							
**SCOPA-SPES**	23.50 (16.5-29.25)	19.00# (16-23)	**0.005**	20.50 (16.25-27.0)	0.316	24.00 (20.0-35.0)	0.462
Subitems							
Motor evaluation	9.00 (6.25-13.5)	7.00# (6.0-10.0)	0.100	9.50 (6.5-12.0)	0.831	12.00 (7.0-15.0)	0.786
ADL	10.50 (9.0-14.0)	9.00# (8.0-13.0)	**0.048**	9.50 (8.0-12.5)	0.944	11.0 (8.0-17.0)	0.495
Motor complications	2.00 (0.0-3.75)	1.00# (0.0-3.0)	**0.008**	1.00 (0.0-2.0)	**0.010**	4.00 (3.0-5.0)	0.680
**SCOPA-COG**	22.00 (14.0-26.75)	28.00# (22-31)	**.000**	27.5 (20.25-30.75)	.0887	20.00 (11.0-27.0)	0.039
Subitems							
Memory	6.00 (4.0-9.0)	8.00# (7.0-10.0)	**0.002**	9.00# (7.0-10.0)	0.604	5.00 (5.0-7.0)	0.073
Attention	3.00 (1.5-4.0)	4.00# (3.0-4.0)	0.067	4.00 (3.0-4.0)	0.762	2.00 (1.0-4.0)	0.457
Executive functioning	9.00 (4.25-10.0)	11.00# (8.0-12.0)	**0.002**	9.00 (4.0-11.0)	**0.015**	9.00 (4.0-11.0)	0.009
Visuospatial functioning	4.00 (3.0-5.0)	4.00 (3.0-6.0)	0.230	5.00# (3.0-7.0)	0.051	3.00 (1.0-7.0)	0.263
**NPI**	2.00 (1.0-6.0)	0.50# (0.0-2.50)	**0.001**	0.00 (0.0-1.00)	**0.046**	0.50 (0.0-1.25)	0.082
Hallucinations (n/%)	10(42%)	6# (25%)	**0.014**	7 (35%)	0.157	5 (38%)	0.753
Depression (n/%)	7 (29%)	4# (17%)	0.083	1# (5%)	0.083	1 (8%)	0.112
Delusions(n/%)	2 (8.3%)	2(8.3%)	1.00	1# (5%)	0.317	0#	0.392
**BDI**	11(9.75-15.0)	7# (6.0-10.0)	**0.00**	9(7.5-12.5)	0.113	12(11.0-14.0)	0.032
**Patients living independently at home** (n, %)	-	20 (83%)		20 (83%)		13 (65%)	

Table 3: Scores are median scores and range IQR25-IQR75, 95% CI.
suggest improvement over time
suggest worsening over time
* Wilcoxon
** Friedman

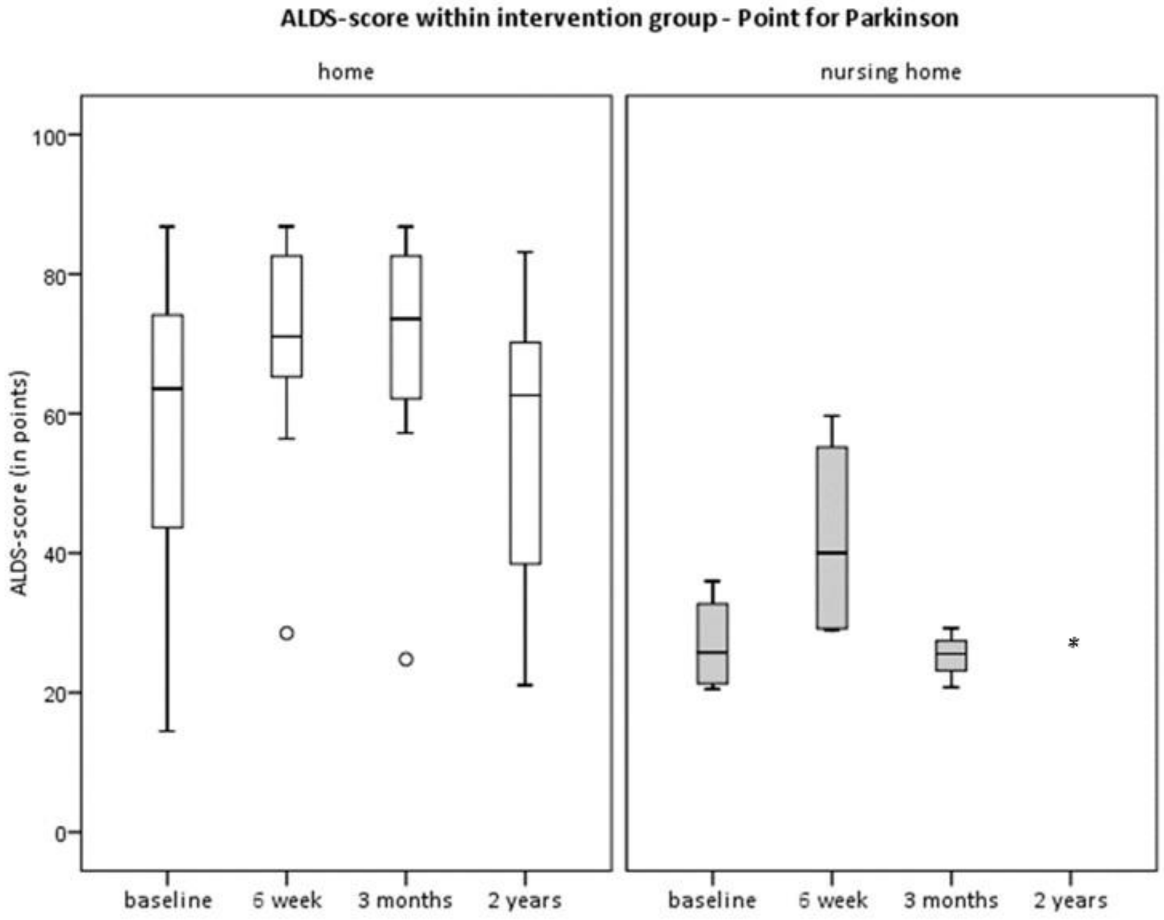

* not enough patients to create a boxplot (n=2).

Figure 1: Sub-analysis: Differences in the median ALDS within the intervention PfP group, comparing patients who could return home (83%) to patients finally admitted to a nursing home (17%).

References Supplements

1. Keus SHJ;, Hendriks HJM;, Bloem BR;, Bredero-Cohen AB;, Goede CJT de;, Haaren M van;, Jaspers M;, Kamsma YPT;, Westra J;, Wolff BY de;, et al. KNGF-richtlijn Ziekte van Parkinson. *Nederlands Tijdschrift voor Fysiotherapie* (2004) 114, (suppl):1-88.

2. Domingos J, Keus SHJ, Dean J, de Vries NM, Ferreira JJ, Bloem BR. The European Physiotherapy Guideline for Parkinson's Disease: Implications for Neurologists. *J Parkinsons Dis* (2018) 8:499–502. doi: 10.3233/JPD-181383

3. Kalf JG, de Swart BJM, Bonnier M, Hofman M, Kanters J, Kocken J, Miltenburg M, Bloem BR, Munneke M. Logopedie bij de ziekte van Parkinson, een richtlijn van de Nederlandse Vereniging voor Logopedie en Foniatrie. Woerden/Den Haag (2008).

4. van der Marck MA, Kalf JG, Sturkenboom IHWM, Nijkrake MJ, Munneke M, Bloem BR. Multidisciplinary care for patients with Parkinson's disease. *Parkinsonism Relat Disord* (2009) 15 Suppl 3:S219-23. doi: 10.1016/S1353-8020(09)70819-3

5. Ingrid Sturkenboom, Marjolein Thijssen, Jolanda Gons-van Elsacker, Irma Jansen, Anke Maasdam, Marloes Schulten, Dicky Vijver-Visser, Esther Steultjens, Bas Bloem, Marten Munneke. 'Ergotherapie bij de ziekte van Parkinson. Een richtlijn van Ergotherapie Nederland.' Utrecht/ Den Haag (2008).

6. van Asseldonk MJMD, Dicke HC, van den Beemt BJW, van den Berg DJ, ter Borg S, Duin GM, Govers SLHM, van Teeffelen JJ, Hoff JI, van Harten B, et al. Richtlijn diëtetiek bij de ziekte van Parkinson. Den Haag (2012).
